# Predicting Regression of Barrett’s Esophagus—Can All the King’s Men Put It Together Again?

**DOI:** 10.3390/biom14091182

**Published:** 2024-09-20

**Authors:** Martin Tobi, Nabiha Khoury, Omar Al-Subee, Seema Sethi, Harvinder Talwar, Michael Kam, James Hatfield, Edi Levi, Jason Hallman, Mary Pat Moyer, Laura Kresty, Michael J. Lawson, Benita McVicker

**Affiliations:** 1 Departments of Medicine, Research and Development, John D. Dingell VAMC, 3636 John R. St., Detroit, MI 48201, USA; 2 Department of Medicine, Wayne State University, 42 W. Warren Ave., Detroit, MI 48201, USA; 3 INCELL Corporation LLC, 12734 Cimarron Path, San Antonio, TX 78249, USA; 4 Department of Thoracic Surgery, University of Michigan, 500 S. State Street, Ann Arbor, MI 48109, USA; 5 Department of Internal Medicine, University of California at Sacramento, Davis, CA 95616, USA; 6 VA Medical Center, Department of Internal Medicine, University of Nebraska Medical Center, Omaha, NE 68005, USA

**Keywords:** Barrett’s metaplasia, p87, spontaneous regression, innate immune system

## Abstract

The primary pre-neoplastic lesion of the lower esophagus in the vicinity of the gastroesophageal junction (GEJ) is any Barrett’s esophageal lesions (BE), and esophageal neoplasia has increased in the US population with predispositions (Caucasian males, truncal obesity, age, and GERD). The responses to BE are endoscopic and screening cytologic programs with endoscopic ablation of various forms. The former have not been proven to be cost-effective and there are mixed results for eradication. A fresh approach is sorely needed. We prospectively followed 2229 mostly male veterans at high risk for colorectal cancer in a 27-year longitudinal long-term study, collecting data on colorectal neoplasia development and other preneoplastic lesions, including BE and spontaneous regression (SR). Another cross-sectional BE study at a similar time period investigated antigenic changes at the GEJ in both BE glandular and squamous mucosa immunohistochemistry and the role of inflammation. Ten of the prospective cohort (21.7%) experienced SR out of a total of forty-six BE patients. Significant differences between SR and stable BE were younger age (*p* < 0.007); lower platelet levels (*p* < 0.02); rectal p87 elevation in SR (*p* < 0.049); a reduced innate immune system (InImS) FEREFF ratio (ferritin: p87 colonic washings) (*p* < 0.04). Ancillary testing showed a broad range of neoplasia biomarkers. InImS markers may be susceptible to intervention using commonplace and safe medical interventions and encourage SR.

## 1. Introduction

The incidence of adenocarcinoma of the lower esophagus has increased 57-fold from 1972 to 2010, outpacing that of squamous cell carcinoma and out of proportion with the relative growth of the US population in that time period [1]. BE is the only histologic precursor for adenocarcinoma of the lower esophagus and a clear genetic predisposition is currently unclear, but in Europe, up to 7% of cases show familial clustering [2]. In view of the alarming statistics, well-intentioned screening programs have been put in place, but efficacy has been questioned [3]. In analyzing an important paper incorporating US and UK screening strategies [4], the data did not fulfill screening guidelines in 39% (UK) and 55% (US), and in those that did, 61% (UK) and 87% (US) did not have symptoms of GERD (gastroesophageal reflux disease), and overall, questions regarding the evidence of the effectiveness of endoscopic screening modalities have been raised [5]. While other minimally invasive screening approaches show promise [4], we suggest that other non-invasive fundamental strategies may prove effective, such as the possibility of inducing regression of BE despite early skepticism [6].

Spontaneous (non-invasive) regression of BE has been described, and we will explain it below.

## 2. Patients, Materials, and Methods

### 2.1. Patients

We initially looked at a small group of patients to characterize the staining distribution of various antibodies and the intensity of labeling using the ABC method (Vector Labs., Newark, CA, USA). Thereafter, using a database of prospectively followed patients at increased risk of colorectal cancer, we endoscopically identified BE and control patients. These patients collected stool samples on which we performed ELISA for p87, used as a denominator for opportunistically obtained blood ferritin levels, yielding the FERAD ratio. We have shown that this biomarker provides an accurate gauge of the innate immune system in viral inflammation [7] and gastric cancer [8]. Embedded were also additional patients who had washings and biopsy obtained at endoscopy and had signed an additional informed consent form. In addition to the former, we used patient FERAD and p38ɣ antibodies (Cell Signaling Technology, Beverly, MA, USA) and the NCM460 stem cell line to assess inflammation and delineate factors leading to disease progression and spontaneous regression of BE. The inclusion criteria were the ability to provide written consent and provide a stool specimen. The exclusion criteria were the presence of previous invasive treatment of BE, or surgery/radiation in the vicinity of the lower esophagus, or the inability to provide consent or a stool sample.

#### 2.1.1. ELISA, Histochemistry, and Western Blotting

Briefly, we performed p87 ELISA on the biopsy on membrane-enriched fractions after Dounce homogenization, followed by sonification on ice and high-speed centrifugations for 5 min and then 10 min for the supernatant. A Bradford or Lowry method protein assay was performed on the resultant supernatant and 5 ug of protein was placed in each well of a 96-well microtiter plate (Nunc, Copenhagen, Denmark), and incubated overnight at 4 degrees centigrade. Plates were blocked for one hour with 5% bovine serum albumin (Sigma, St. Louis, MO, USA), washed 3 times with phosphate buffered saline with Tween-20, and incubated with the primary Adnab-9 monoclonal antibody (Dako Inc., Carpinteria, CA, USA), at a concentration of 1:500 on half the plate and UPC10 1:500, an irrelevant antibody (Sigma, St. Louis, MO, USA) with the same isotype as the primary antibody, for one hour at 37 degrees, as a negative control. Positive control was adenoma extract, as derived above. Secondary anti-mouse antibody linked to alkaline phosphatase was incubated and washed as above along with PNPP (p-nitrophenol phosphate) substrate (Sigma, St. Louis, MO, USA) to generate color at 20 mins, and Optical Density was measured at 405 nm (Titertek Multiscan, Flowlabs, McClean, VA, USA). The control readings are subtracted from the test sample, yielding the final result (OD-background).

The immunohistochemistry kit (Vector Labs), was also used according to the manufacturer’s directions with Adnab-9 as the primary monoclonal. No microwave exposure was used to enhance antigen visualization. The kit was used to stain EGD-biopsy tissue from approximately 70 patients enrolled and confirmed to have BE or GERD by our staff pathologists, and patients with BE at the GEJ were labeled with a medley of monoclonal antibodies (see Table 1). The stains were graded from 0 to 5+, as reported previously [8].

Western blotting on 9.6% SDS-polyacrylamide gels (Sigma) was loaded at 10 ug/lane on SDS-gels and electrophoresed according to the manufacturer’s directions and transferred to PVDF (polyvinylidene difluoride) membranes (Kirkegaard & Perry, Gaithersburg, MD, USA) antigen-visualized using the above sequence of antibodies using the ABC kit (Vector Laboratories) and relative mobility used to compute molecular weight. The NCM460 cell line was gifted by INCELL Corporation and is a group that is specialized in proprietary medium. The cells were grown at the period of exponential growth (70–80% confluence) and were serum-starved for 48 h. The medium was then aspirated and replaced with acidic medium (pH 4) for 5, 10, 15, 30, and 60 min. Following treatments, the cells were harvested and twice washed with PBS. The cells were then extracted with RIPA buffer containing phosphatase and protease inhibitors. A total of 25 µg of each sample was loaded onto a 10% SDS gel, electrophoresed, and then transferred onto nitrocellulose membranes and blotted with different antibodies (ppERK, pp38ɣ, and pp38 αβ).

Since there is an association between BE and *H. pylori* [9] and diabetes mellitus [10], we also looked for an increase or decrease, respectively, to support these notions, particularly when prospective *H. pylori* infection might mitigate the severity of BE, leading to regression, while diabetes may have the opposite effect.

#### 2.1.2. Immunohistochemistry

One group of 34+ patients had cold forceps biopsies of the GEJ whilst undergoing esophagogastro-duodenoscopy (EGD) for various clinical indications as per the request of the primary care provider. All of these studies were performed under the supervision of an attending gastroenterologist and a standard report describing the clinical findings was entered into the patient database reference folder which was later accessed. We graded discernible staining, and thereafter, a pathologist determined the presence of BE on H&E section review using standardized criteria compared to native antigen anti-CDX2 antibody background immunostaining. For reproducibility, multiple sets of slides were labeled and the mean was presented as a percentage.

The monoclonal antibodies that were used are reflective of the repertoire used at the time of inception and those in current use to assess antigenic association with neoplasia and inflammation; the classical hallmarks and outcomes are tabulated above.

#### 2.1.3. Permissions and Approvals

The study was approved by the IRB (institutional review board) and Human Investigation Committee of Wayne State University. The approval numbers are #070700MP4F and #H 09-62-94, and the approval dates are 13 September 2006 and 17 August 2000, respectively. All patients gave informed consent.

#### 2.1.4. Statistical Analysis

The statistical analysis was performed used an online statistics package (Vassar Stats http://vassarstats.net/ and last access was 22 May 2024). The normality of values was tested using the online Kolmogorov–Smirnov calculator (https://www.socscistatistics.com/tests/kolmogorov/, accessed on 5 December 2023).

## 3. Results

### 3.1. Tabulated Results

#### 3.1.1. Schematic Summaries and Actual Photomicrographs of Monoclonal Antibody Staining

We used the following Table 2 and Table 3 markers to get an idea of how antigens are expressed in BE and squamous epithelium in both BE and non-BE; the latter would approximate the staining that would be expected to occur in regressed epithelium.

**Figure 1:**
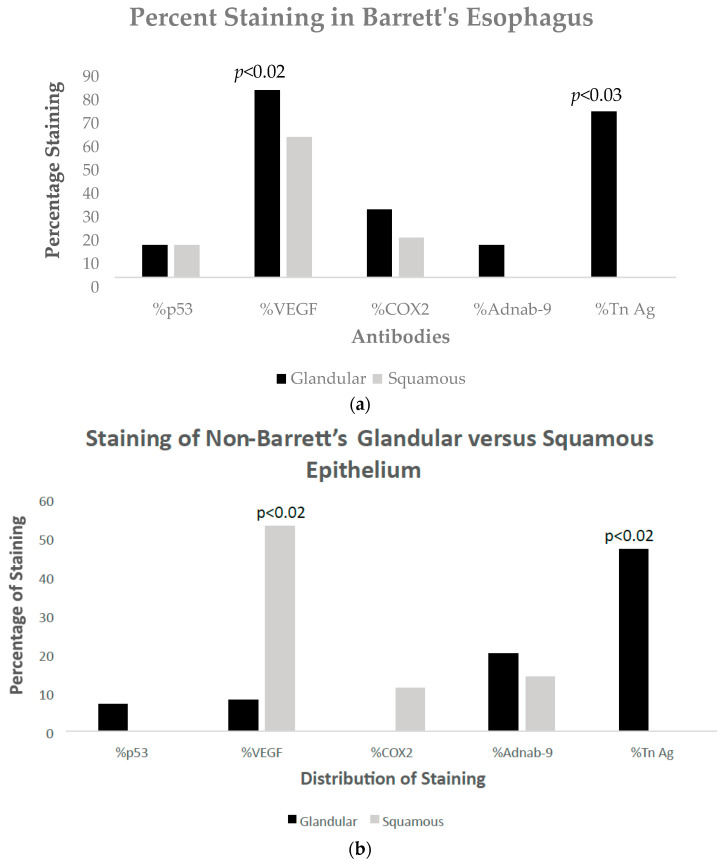
(**a**) Bar diagram of percentages of staining with various antibodies in glandular versus squamous epithelium in Barrett’s esophagus. (**b**) Bar diagram of percentages of staining with various antibodies in glandular versus squamous epithelium in non-Barrett’s esophagus.

**Figure 2:**
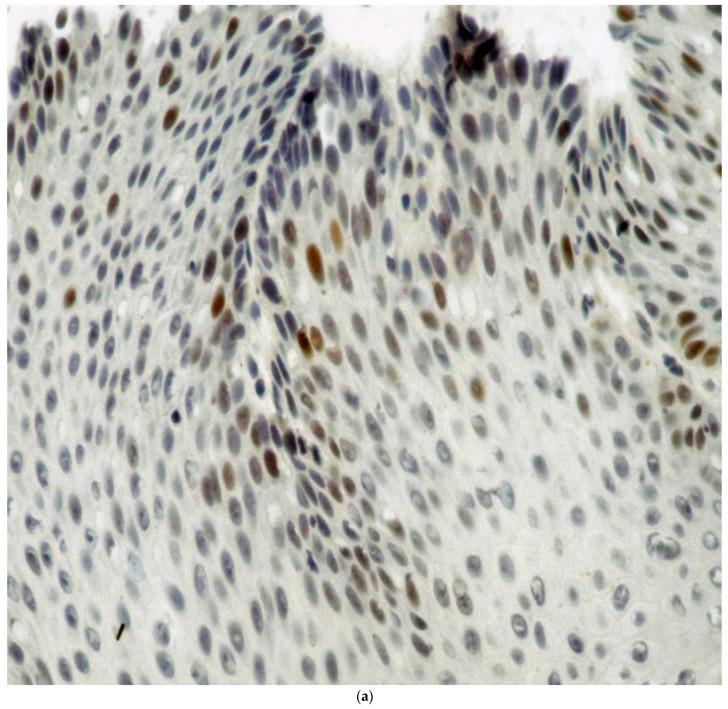

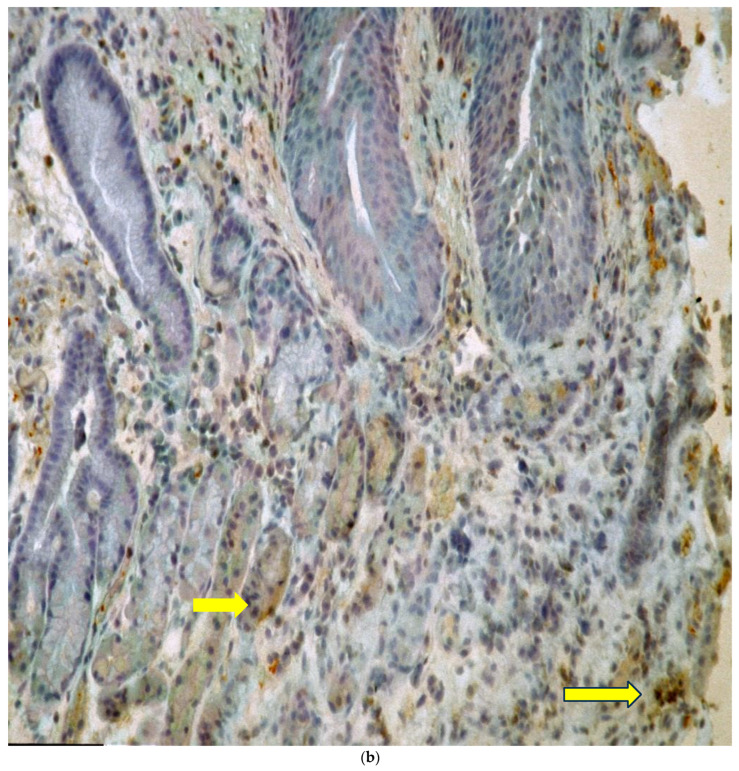

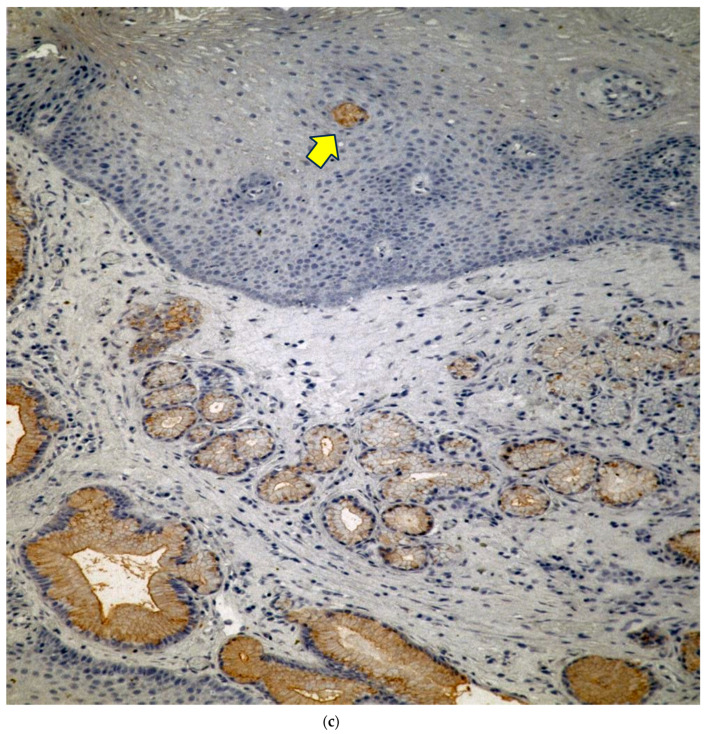

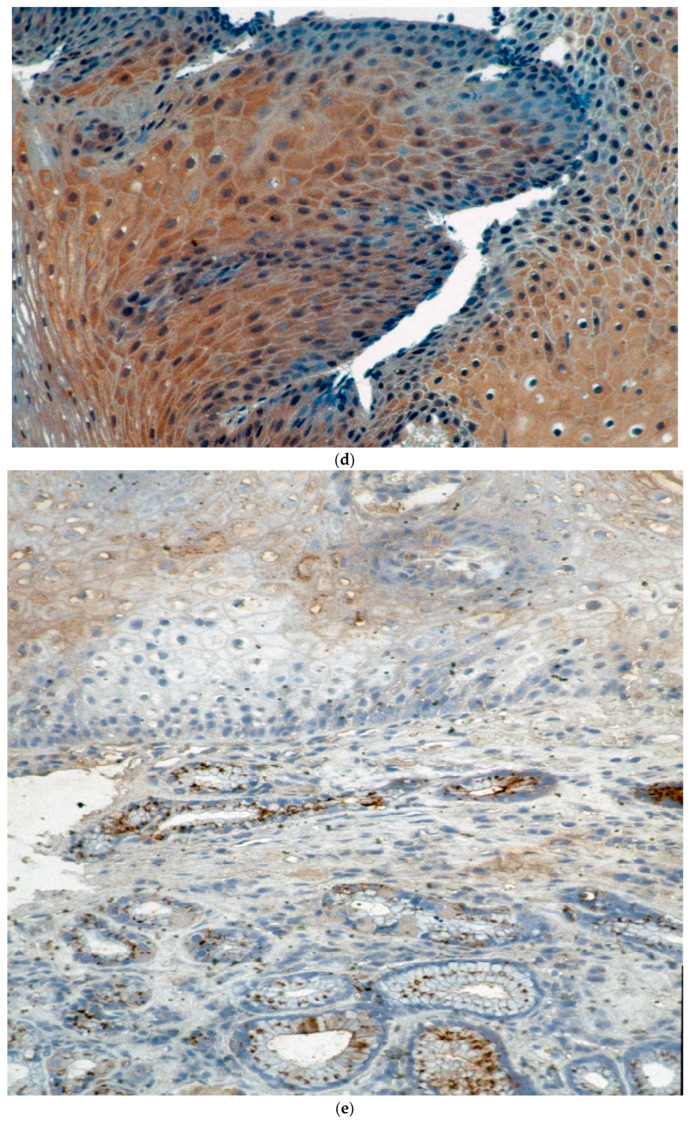

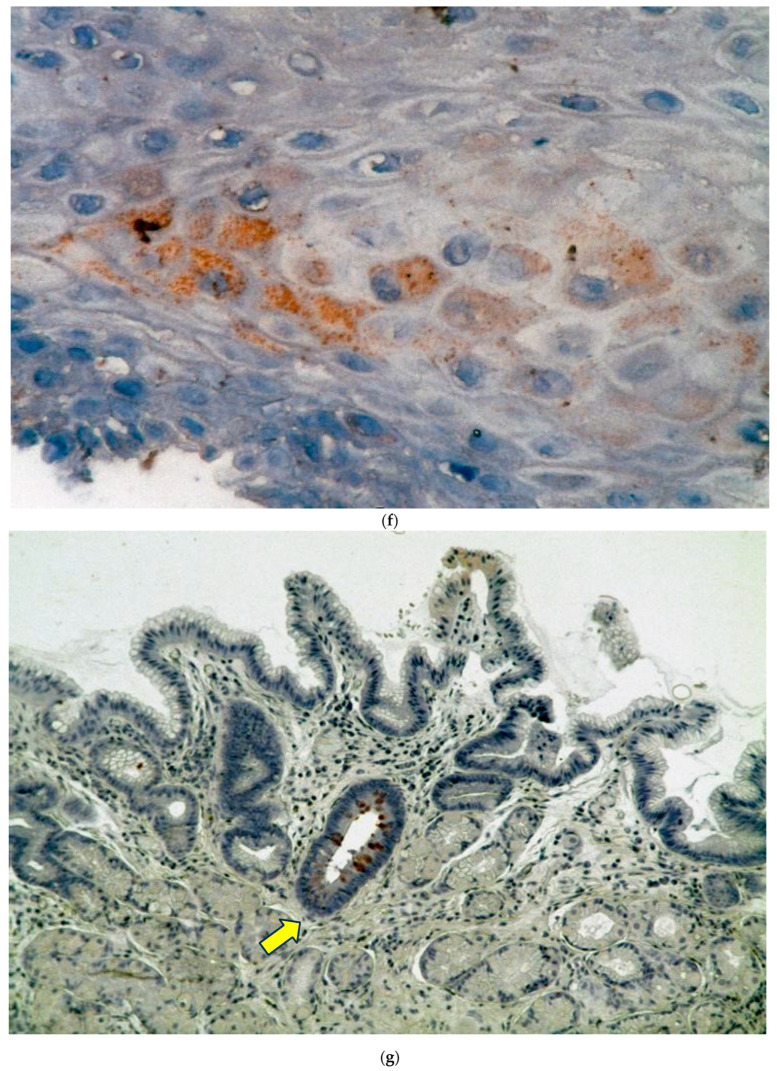

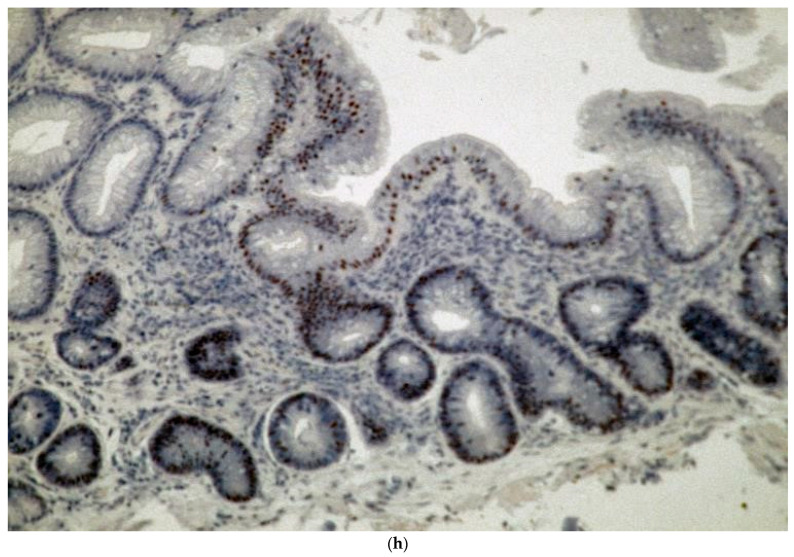
(**a**) Percentage staining of squamous nuclei in a Barrett’s esophagus patient with p53 nuclear staining as evidenced by the brown nuclei. Magnification 360×. (**b**) VEGF staining in a glandular section of a specimen taken from a patient with Barrett’s esophagus. (**b**) shows moderate brown VEGF staining in the glandular epithelium more localized in the cytoplasm (arrows indicate glands and staining). Magnification 50×. (**c**) Cox2 staining is seen from a section taken through the GEJ in a section taken from a patient with Barrett’s esophagus. Magnification 50×. (**d**) COX-2 staining in the squamous epithelium is moderate diffuse and cytoplasmic. Magnification 130×. The intensity of stain was 3+ in the areas on the left and 1–2+ on the right. (**e**) Adnab-9 staining of a section taken from the GEJ of a patient with confirmed Barrett’s esophagus. Esophagus. Magnification 50×. (**f**) shows Adnab-9 labeling of squamous cells in a patient with Barrett’s esophagus focally with reticulated cytoplasmic staining at a higher power. Magnification 360×. (**g**) Tn staining of glandular mucosa in a patient with Barrett’s esophagus. Magnification 50×. (**h**) Intense nuclear CDX2 staining is seen in this section of Barrett’s epithelium and involves most of the glandular epithelium in this section. Magnification 125×.

The results of the staining distribution in glandular and squamous epithelium can be seen in Barrett’s Figure 1a and in non-Barrett’s Figure 1b. VEGF is significantly maximal in non-BE squamous epithelium and is probably a GERD effect, as can be seen in Figure 2f below and the resultant comments and references cited [11]; see Section 4. Forty-three specimens from 29 patients were stained with p53.

Figure 2a–h shows the staining with the monoclonals.

Approximately one-third of nuclei are heavily stained, suggesting that abnormal p53 is present from a section of Barrett’s squamous epithelium, suggesting a pre-malignant phenotype. The intensity of the stain in the heaviest stained nuclei was 4+.

Forty-nine specimens from 30 patients were stained with VEGF. The intensity of the stain was 2–3+ in the areas indicated by the arrows.

In Figure 2c, most of the Cox2 staining is confined to the epithelium of dilated glands seen to the right-hand side of the photomicrograph. The yellow arrow shows a focus of staining in the stratified squamous epithelium of the lower esophagus in center field and is likely to be non-specific staining of a rete peg, which is usually associated with false positive stains. The intensity of the stain was 2–3+ in the area indicated by the arrow, which compared favorably with the intensity of the glands in the lower foreground.

Labeling shows intense cytoplasmic staining of the Golgi apparatus in the glandular mucosa in the lower foreground of the photomicrograph and affects most of the glands. This likely represents a reaction of the innate immune system, as this antigen is usually released by Paneth cells, which are part of this system. The faint staining in the upper section of the photomicrograph at the intercalation of squamous epithelium is actually glandular epithelium. The heaviest intensity in the glandular section below is 4+.

The squamous epithelium shows intense brownish red but focal cytoplasmic labeling, suggesting an InImS similar to the glandular component in the above Figure 2e, and is 3+ in the heaviest intensity.

This section of a photomicrograph cut through a glandular section of BE with focal globular dark Tn cytoplasmic staining. The very dense In cytoplasmic staining of the central gland suggests intense focal staining with the anti-Tn antibody. The yellow arrow indicates the gland in which a number of cells express the Tn antigen. The heaviest intensity of staining in the gland highlighted by the arrow is 5+.

The nuclei in this CDX2 stained glandular epithelium show mainly nuclear staining.

Having noted the diversity in staining and the outcomes in both inflammatory and neoplastic biomarkers, we sought to expand the sample size by selecting from an extensive database of patients at increased risk of colorectal cancer where additional parameters of Barrett’s related changes had been noted. Non-invasive follow-up of ~2293 patients, with 451 patients having undergone EGD, we found approximately 65 BE patients, where 40% had inflammation and 60% were noted as non-inflamed. The original total had been 83 BE patients, but since some data were unavailable or inconclusive, we were only able to endorse 46 patients, finding that 10 had regressed and 36 remained stable over time. Twenty-one specimens from 29 patients were stained with CDX2. The heaviest intensity in the gland nuclei is 4–5+.

#### 3.1.2. Tabulation and Correlation of the Effects of Inflammation in Barrett’s Epithelium

Table 4 indicates a significantly lower association of inflammation with both serum and urinary creatinine, but this may not correlate with renal function, as glomerular filtration rates were not available for comparison. Significant correlation with inflamed BE is evident with increased alcoholic intake but has an opposite effect with overweight patients with inflamed BE, and the cause of this association is obscure for now. Dysplasia of BE is higher in inflamed BE, as might be expected, but there is no significant difference. In patients with normal mucosa, AA with no inflammation is over-represented, which would be expected, as BE is less commonly found in AA.

Additionally, trends for BE vs. normal epithelial (NE) groups were seen with p87 Western blots (7 vs. 14.7% res-*p* = 0.16): quantitative alcohol intake, 53% vs. 39%—*p* = 0.07; predominance of males, 95 vs. 89%—*p* = 0.15; PPi exposure, 41 vs. 30%-0 = 0.18. Trends for BE with and without inflammation were seen for FERAD ratio (ferritin/stool p87), 21,477 ± 37,197 vs. 20,928 ± 66,593-*p* = 0.14. Comparing BE with inflammation and NE, trends were seen for African Americans (AA), 10/25 (40%) vs. 8/38 (21%)—*p* = 0.096; quantitative alcohol (Quan EtOH), 5/16 (31%) vs. 103/189 (55%)—*p* = 0.12. Trends of BMI levels for BE with no inflammation and NE with no inflammation were 22/33 (67%) vs. 81/151 (54%)—*p* = 0.18, and likewise, there were other trends for PPi (proton pump inhibitor) exposure, 10/23 (44%) vs. 29/102 (28%)—*p* = 0.2; ACE exposure, 9/26 (35% vs. 56/104 (54%)—*p* = 0.1; and CCB (calcium channel blocker) exposure, 6/19 (32%) vs. 50/90 (56%)—*p* = 0.057 (by Pearson’s Chi-square).

**Figure 3:**
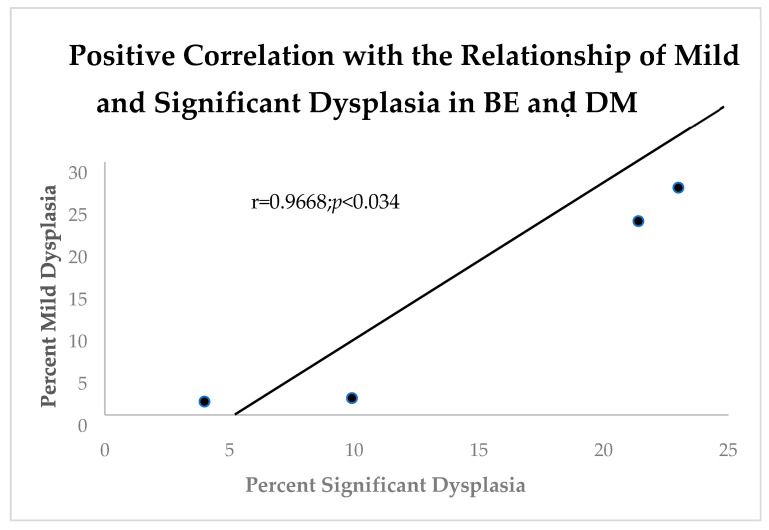
Linear correlation diagram shows an expected positive relationship between mild and significant dysplasia in BE Patients with diabetes mellitus.

It is well known that diabetes mellitus (DM) causes generalized increased inflammation [10]. In order to contrast BE and the effects of DM where the vast majority were type 2, we compared the effects of DM with mild and severe dysplasia (Figure 3).

**Figure 4:**
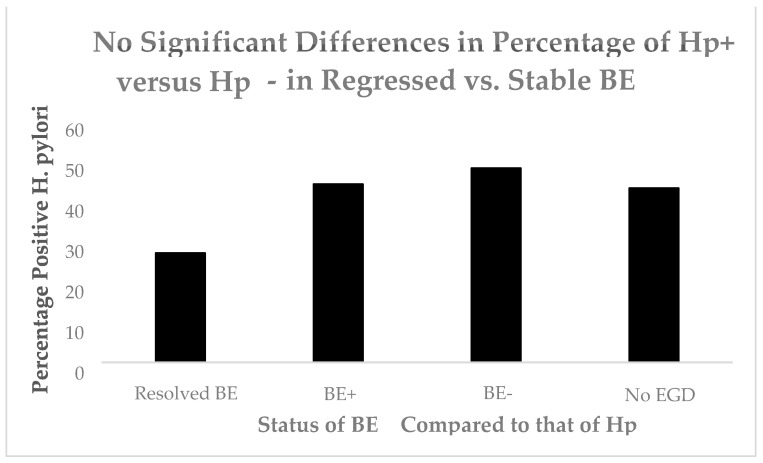
A bar diagram showing no significant differences of Hp in BE.

Other have confirmed the relationship of BE and DM [12]. Having shown an effect of DM, we turned to another upper gastrointestinal tract purveyor of inflammation, *Helicobacter pylori* (Hp). Figure 4 shows no significant effect of Hp in BE.

**Figure 5:**
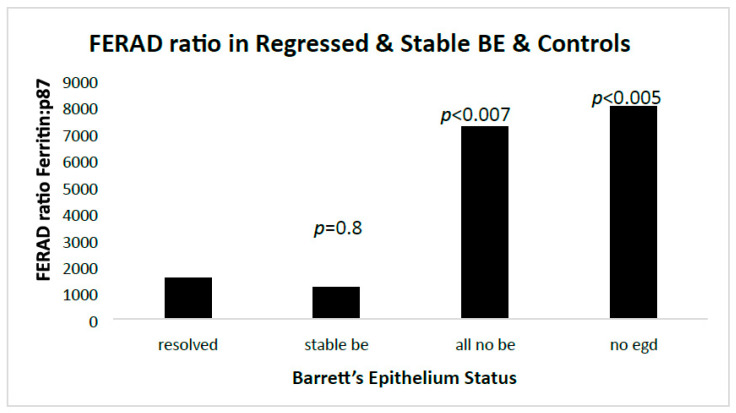
A bar diagram showing the proportional effects of the FERAD ratio in those with and without BE.

Since, thus far, only DM had an effect on inflammation and neoplasia, we explored the role of an InImS biomarker, the FERAD ratio, and found significant effects only between patients without BE, and none between regressed and stable BE (Figure 5).

The non-EGD group included a large group of patients that were used as an undefined group for comparison with the refined groups. All groups were compared to the regressed BE patients.

**Figure 6:**
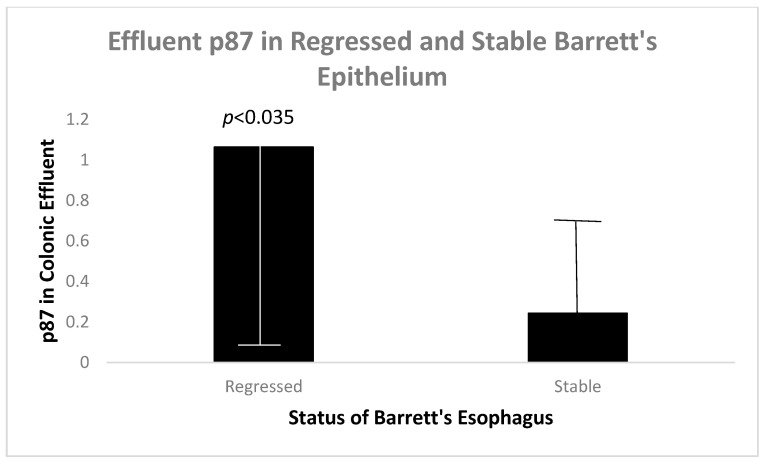
A bar diagram showing higher mean p87 in the colonic effluent of regressed Barrett’s epithelium patients as opposed to stable patients.

Since we have shown that p87 correlates with inflammatory and preneoplastic states [7,8], we looked at p87 in colonic washings (effluent) as another biomarker of the InImS (Figure 6).

**Figure 7:**
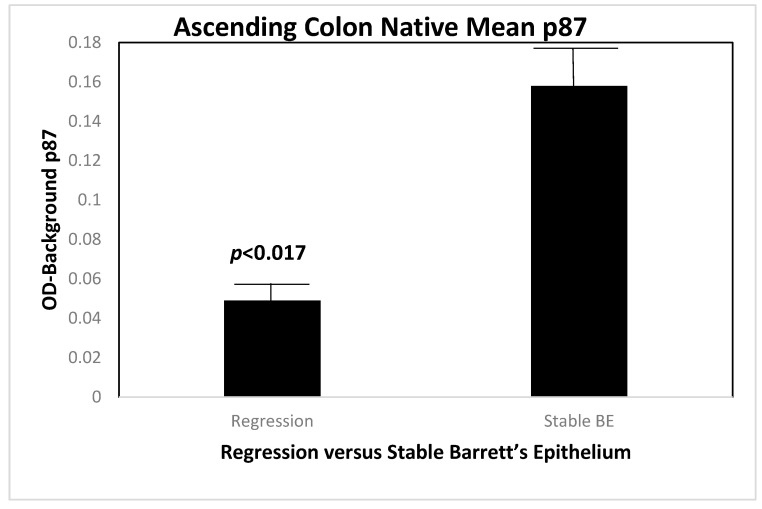
A bar diagram showing the higher level of native p87 in the ascending colon of patients with regressed BE as compared to those with lower levels.

It was of much interest to reveal the source of the p87 in patients with regressed versus stable BE. We therefore contrasted the origins of native (non-fixed) p87 throughout the six regions of the colon in these two groups of patients, and the result can be seen in Figure 7 in biopsies taken from the ascending colon.

**Figure 8:**
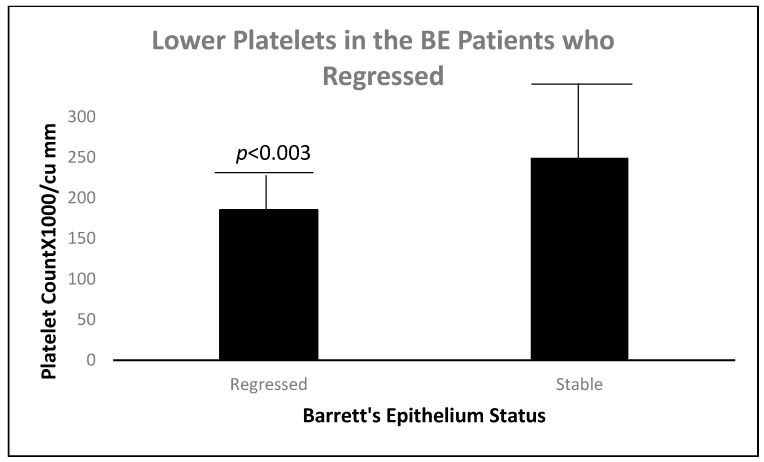
A bar diagram showing a significantly lower platelet count in the patients with BE regression.

Figure 8 shows a statistically significant lower mean expression of native (unfixed) p87, in a location that is usually rich in Paneth cells. This suggests greater shedding of p87 from this area of the colon in patients with BE regression, and explains the higher p87 content in colonic washings (see Figure 6). In view of factors that drive inflammation [13], we looked at platelet levels in regressed and stable BE. We were able to show a strong relationship between low platelet counts and regression of BE.

In view of the cited regression of BE by PPi medications [14], we investigated the proportion of BE patients taking PPi and found that the proportions of BE regression patients were equivalent to those with stable BE (37.5 versus 38%), but we did not quantify the doses.

**Figure 9:**
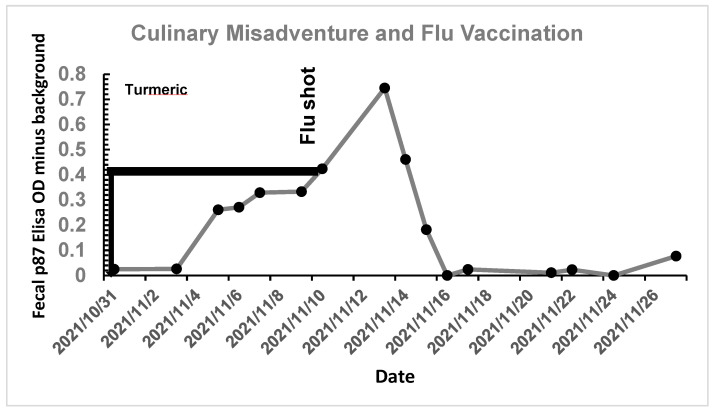
Effect of turmeric ingestion, followed by influenza vaccination, on fecal p87.

Importantly, it must be shown that the p97 can be manipulated to bring both stool p87 and FERAD ratios in order to achieve regression inter alia with anti-platelet medications. Figure 9 shows the effect of an undisclosed dose of turmeric on stool p87 of a 68-year-old otherwise healthy male, followed by a hospital-mandated flu vaccination.

An undisclosed quantity of turmeric in vegetable broth was ingested for 11 days with a steady increase in fecal p87. This was followed by an influenza vaccination, resulting in a further increase to a peak of OD-background of 0.75 and then a decline to baseline at about 2 days thereafter. Turmeric therefore likely increases p87 and can be used to stimulate the InImS with little financial cost to the patient. However, it is also important to have a strategy that will enable suppression of p87 and the InImS.

**Figure 10:**
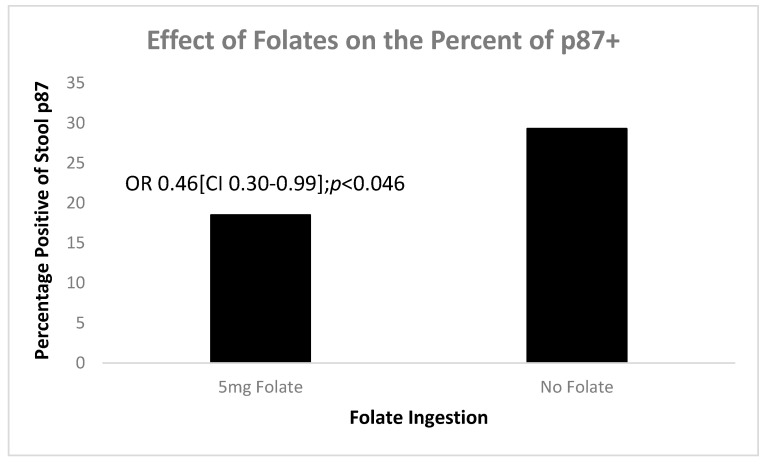
A bar diagram shows that a daily dose of 5 mg folate significantly reduces stool p87.

Our database revealed that patients were taking varied amounts of folic acid and we were able to measure p87 in 20 patients taking 5 mg versus 23 of those who were not. Figure 10 shows that folic acid significantly reduced positivity of p87 (OD-background > 0.05) compared to those not taking folates.

These data support our supposition that p87 can be manipulated in order to encourage regression of BE. We now used in vitro testing to investigate the effects of low pH on cell lines.

#### 3.1.3. Investigation of the Cell Line MAP Kinase (MAPK), Biomarker, and Adhesion Molecule Response to Exposure to Normal and Physiologically Low pH

**Figure 11:**
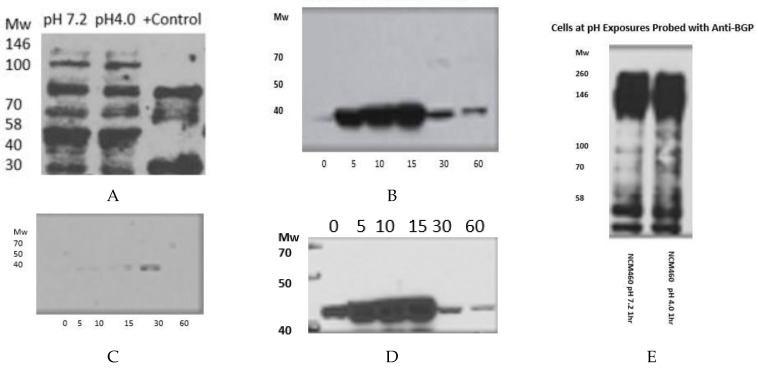
Adnab-9 is pH Indifferent (**A**). Early Response of pp38ɣ (**B**). 30 min pp38 Mab response (**C**). Anti-ppERK Response (**D**). Western blot of NCM460 cell lines exposed to different pH levels for 1 h, stained for BGP using the CEACAM1 monoclonal (**E**).

Investigation of the Effect of Normal and Physiologically Low pH Exposure on the Expression of the MAP Kinase (MAPK) Biomarker and Adhesion Molecule Response in the NCM460 Cell Line (Figure 11, Original images can be found in Supplementary Materials).

In Figure 11A, showing incremental time exposures to pH 4, expression at low pH does not appear to affect p87 expression.

The physiological pH is 7.2 and the acidic pH is 4, and these exposures were all used for Figure 11.

Figure 11E shows that little difference is discerned between the blots at normal or low pH levels.

**Figure 12:**
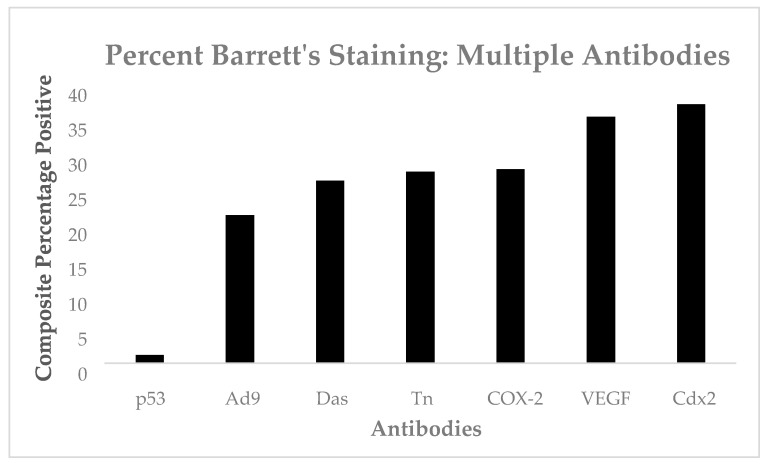
A bar diagram with percentage staining of antibodies used.

We do have cumulative data from our retrospective series, but there were no statistically significant differences between the antibodies and only one case stained with p53, as seen in Figure 12.

## 4. Discussion

Dutch researchers were amongst the first to recognize that regression of BE may reduce the risk of cancer [15]. This was a prospective, double-blind, randomized controlled study that used omeprazole 40 mg twice daily to suppress acid secretion to 0.1% of initial secretion and showed a significant regression of BE on repeated endoscopic biopsy follow-up in 68 patients only with the omeprazole treatment with no effect with ranitidine 150 mg bid as the control, over 2 years. In another prospective, longitudinal study, seven of ninety-nine patients followed for 24–106 months showed complete regression of BE. There was an average endoscopic surveillance of 1.3 endoscopic examinations for each year of the study, with continuation of anti-reflux medications. The BE was confirmed with Lugol’s stain and the length of the BE noted [16]. Logistic, multiple regression showed that absence of a hiatal hernia appeared to be the only factor influencing the BE regression. Stepwise regression analysis also implicated length of the BE and absence of a hiatal hernia. A Korean publication [17] studied immunohistochemistry in 25 patients and found that 11 (44%) regressed and suggested an association between regression and lower grade of BE, less KI67, CDX2 expression. Retrospective data from the US [18] looked at 1382 patients with reflux who experienced regression in 505 patients (37.6%). While omeprazole intake was unassociated with regression, vitamin D was significantly increased, while the length of BE was significantly shorter. Interestingly, one patient who regressed was found to have dysplasia, which is unusual but soberly suggests that not all patients who regressed will be immune from neoplasia. No individual data for this patient were provided. However, it did confirm a statistically lower prevalence of hiatal hernia in the patients with BE regression (55.8 vs. 61.8%, respectively). BE regression has also been shown to be a consequence of gastric surgery [12].

The factors in regression mentioned above are all local phenomena and do not relate to a global picture. It has been postulated that the cells of BE emanate from different sources, even unexpected, such as the bone marrow [19]. Given the scope of BE and the likely interaction of this disease with the innate immune system, we expanded this paper to include both traditional laboratory investigation and novel, emergent innate immune system markers [7,8]. Two papers published in 2020 have shed some light on the relationship of the innate immune system in esophageal adenocarcinoma (EAC) and BE, the first by Gokon et al. [14], with immunolocalization in 83 EAC patients compared to 13 controls with BE. Abundant Fox3+ lamina propria lymphocytes (LPL) were found in the EAC patients’ BE sections with a tendency to be linked to p53 expression, and CD8 LPLs in the BE were associated with a worse prognosis in the EAC patients, suggesting a pro-neoplastic immune microenvironment. The second paper by Lagisetty et al. used RNA sequencing analysis, showing that an incremental increase in a subset of cytokines and chemokines, such as IL-6 and CACLB, was characteristic of progression from BE to EAC and implicated M-2 macrophages, pro-B cells, and eosinophils by xCell deconvolution analysis [20]. Compared to BE, EAC microarray immunostaining showed a paucity of immune cells in the face of PD-L1 predominance. We were most interested in the innate immune system as evaluated by a biomarker we termed the FERAD ratio, which uses blood ferritin as the enumerator and fecal Adnab-9 monoclonal binding as the denominator and p87 as the antigen recognized by Adnab-9. We have found this Yin and Yang ratio to be useful in predicting susceptibility to and severity of COVID-19, also correlating with the established absolute neutrophil:absolute lymphocyte count (NLR) [7], and it is also a prognostic marker in gastric adenocarcinoma [8]. We have also applied this biomarker to investigate aspects of BE regression [6], and if we can increase our understanding of the role of the innate immune system in inducing BE regression, we could conceivably eliminate the need for invasive interventions and morbidity. To the best of our knowledge, no workers in the field have attempted this strategy.

The debate regarding the source and role of BE continues unabated, and recently, an exhaustive and thorough study using transcriptomics and genomic techniques concluded that the MB arises from undifferentiated gastric cardia via c*MYC* and *HNF4A* transcriptional pathways and that EAC arises from undifferentiated Barrett’s cell types with no identifiable precursor [21]. Previously, p53, a nuclear transcription factor controlling proliferation and genetic stabilization, was a prime candidate and was the most prominent change seen in a large number of cancers, but very early on, a lack of correlation between p53 expression and tumor stage suggested that p53 protein overexpression is an early event in these tumors [22]. VEGF plays a crucial role in angiogenesis of many solid malignancies and, together with p53, correlates with the grade of the malignancy, and VEGF might play a role in the early stage of esophageal carcinogenesis [23]. COX-2 enzyme is often over-expressed in BE and esophageal adenocarcinoma as well as SCC, and one study showed clear COX-2 expression in the epithelial cells in Barrett’s metaplasia, which confirms elevated expression in adenocarcinoma, and it showed that the elevation in expression occurs in the progression from LGD to HGD [24]. Another study concluded that acid increases COX-2 expression through activation of the MAPK pathways, suggesting that acid reflux might promote carcinogenesis in Barrett’s esophagus [25,26]. While p87 is the antigen recognized by the Adnab-9 monoclonal, it is present in premalignant conditions [8] and appears to be part of the InImS and herein seems to play a role in the regression of BE, as the focal nature of staining is proportionate to that shown by Nomura et al. [27] and other authors, who speculate that regression may be associated with low MPC expression. In this study, CDX2 has reasonable sensitivity for BE, whether considering patients or sections, but lacks specificity in individual glandular sections and is thus inferior to a pathologist’s determination of BE. However, some do believe that in the change from squamous to intestinal phenotype, Cdx-2 may play a role as a premalignant marker [28].

Although reports of BE regression are highlighted in the introduction, they are not representative of the entire experience of regression, and while some appear to be successful in cases of Roux-en-Y gastric bypass, others could report only a single case in 1988 [29]. Later reports publicized bypass surgical response in the region of 62–64% in short and long BE segment after a mean of 47 months’ follow-up, but no effect on five patients with “extra-long” BE segments; only one of seventy-eight patients had a finding of low-grade dysplasia [30].

The results of the Western blotting show that acid has little effect on p87 or CEACAM1, and we have previously shown that there is a direct correlation between the two in primates [31]. Low pH causes phosphorylation of p38 by its upstream inflammatory congener, ERK [32]. We used the human colon mucosal cell line as a quintessential example of abundant mucin globules, which approximates the hallmark globules of BE. Seg1 has long been used as an alternative cell line to study BE, but in reality, it is more akin to lung adenocarcinoma, and its use should be avoided [33]. FLO-1, OE33, and OE21 cell lines, however, have a similar reaction to NCM360 cells. Although we did not discuss CDX2 or mAbDas-1 results, we had a small prospective series and had similar staining characteristics, with 46% positive for mAbDas-1 and 42% positive for CDX2. Of interest is that the BART-1 BE cell line, after prolonged exposure to bile salts and acid, can transform its phenotype and adopt colonocyte cellular characteristics [34], somewhat akin to the NCM360 cell line.

In terms of the antigenic landscape, the consistent factor for neoplastic progression of BE is abnormal p53 IHC staining, and a recent study with p53 staining in two cohorts, in 561 patients with and without progression, regardless of the diagnosis of dysplasia in retrospective and prospective cohorts [35], used methods similar to those used in our study. The latter had two pathologists, while theirs had a total of forty-one. However, our IHC was performed by a very experienced PhD in a single study, as opposed to three centers in their study, which may have possibly led to greater heterogeneity. That said, their study was a well-performed study, much broader in scope. p53, COX2, and Tn [36] have a possible role, but they have not been simultaneously studied in the Barrett’s transition. A publication that looked at VEGF found staining in 52% of moderate GERD and found a significant correlation with SSBE [13]. BE Length in COX-2 staining did not correlate. That said, COX-2 is putatively implicated in EAC, and accordingly, aspirin has been shown to reduce the cancer risk. As expected, COX-2 significantly correlated with inflammation but not progression [36]. The Tn antigen is an O-glycosylated glycan often found in cancer tissues, with complex amino acid moieties [37,38], and in Barrett’s cancers, Tn staining is found to correlate with depth of EAC invasion. Autocrine-secreted VEGF is universal in BE and dysplastic BE and VEGF receptors stimulate an ERK pathway [31], as we have shown in our Western blot NCM360 cells. In this study, CDX2 has reasonable sensitivity for BE whether considering patients or sections, but lacks specificity in individual BE sections and is thus inferior to a pathologist’s determination of BE. In Figure 2h, the general nuclear staining has been described in both non-dysplastic and dysplastic Barrett’s mucosa, and the global nuclear staining is seen, but the intensity increases in dysplastic epithelial nuclei [26].

**Figure 13:**
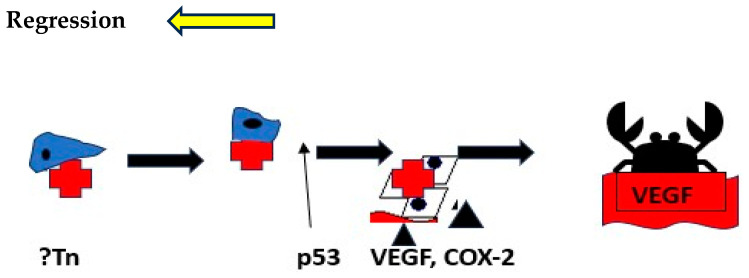
Stylized putative pathway leading to EAC in BE and regression.

The p87 antigen recognized by the Adnab-9 monoclonal stains glandular BE, and regression is associated with gut-secreted p87, the source of which appears to be the rectum. In primates, p87 correlates with CEACAM1 [31], and the latter is a downstream effector of VEGF [39]. This concordance may be associated with BE progression. mAbDas-1 shares some staining characteristics with Adnab-9 in both BE [40] and intraductal papillary neoplasms of the pancreas [41]. All patients participating in the immunohistochemistry assays are described in Section 2.1.2. We offer Figure 13 as a stylized progression model thatwe have termed the MeDyCan pathway to neoplasia. In Figure 2f of Adnab-9 staining, the position of the eccentric nuclei with a basilar position suggests that all the cells with this configuration are in fact metaplastic Paneth cells (MPCs), known to be expressed in Barrett’s esophagus [42]. It has been shown in animal experiments that bile acids and acidic pH can induce squamous MPC [43]. Furthermore, Nomura et al. exposed a squamous cell in vitro culture to alpha-defensin-5, a Paneth cell secretion, and the expression of E-cadherin was reduced [27]. They suggested that cell–cell interaction may be reduced as a consequence, possibly leading to progression, but stopped short of assigning a neoplastic role.

The factors may allow BE regression by actors of the InImS and anti-inflammatory/antiplatelet medicine, at its earlier stages. Further studies will be needed to confirm this scenario. Tn initiates, and p53 indicates the point of no return and the crab, EAC.

## 5. Conclusions

Regression of BE appears to be related to lower hematologic platelet levels, InImS Paneth cell shedding, shorter length of Barrett’s epithelium, and lesser inflammation, younger age, and lower BMI values. Not all of these parameters are actionable, but some are, namely antiplatelet medications and NSAIDs, the latter being the least toxic, and it might also reduce inflammation. The InImS can increase activity by using turmeric or reduce p87 by using folates. Weight loss programs are on offer to veterans seeking to lose weight and reduce BMI. Certainly, a prospective, randomized, double-blind trial using these relatively innocuous measures may aid BE regression. From earlier studies, intensive PPi therapy has been shown to be effective. Omeprazole also has anti-neoplastic activity demonstrated in CRC [44] and breast [45] cancer cells, and we have previously shown that the growth of an aggressive neuroendocrine cell line NCI-H716, originally of colorectal origin, was curtailed significantly by omeprazole, in a milieu of gastrin added to the tissue culture medium [44]. The Adnab-9 binding to p87 was low in Barrett’s esophagus, regardless of regression or stability (Figure 1a,b), and was the lowest of all antibody labeling aside from p53 (Figure 12). The overall setting of the InImS, as gauged by the FERAD ratio, was also low (Figure 5), suggesting that the progression of Barrett’s neoplasia is “under the radar”. We have shown this proclivity in both infections (COVID) and gastric cancer [7,8]. Other moderating factors, such as H. pylori, are not operative (Figure 4). However, regression of Barrett’s epithelium does bear associations with increased effluent p87, presumably due to the reduced expression of native p87 in the mucosa of the ascending colon (Figure 6 and Figure 7). While p87 is found in both glandular and squamous portions of Barrett’s epithelium, this would appear to be a reaction to the effects of inflammation (Figure 2e,f), whereas the reported Paneth cell metaplasia appears to have an identical distribution at the base of the glandular epithelium and exhibit the morphological appearance of such cells in the normal small bowel [46]. Although there are contradictory reports about the efficacy of PPi in preventing cancer [47,48], they can be part of the armamentarium in the kings’ stable that we have outlined above, to affect regression on Barrett’s epithelium, and may also reduce EAC incidence by a minimum of 19% [47]. The role of diabetes adding to inflammation awaits elucidation [49]. 

## Figures and Tables

**Table 1:** List of monoclonal antibodies used for immunohistochemistry of chronic GERD.

Monoclonal Antibody	Biomarker	Source
Anti-p53	Early de novo; late ACS *	Dako Inc.
Vascular Endothelial Growth Factor	Angiogenesis	PharMingen (San Diego, CA, USA)
Adnab-9	ACS	Dako Inc.
COX-2	ACS	Cayman (Ann Arbor, MI, USA)
Anti-Tn	IBD’ de novo	Dako Inc.
Cdx-2	IM marker ^	Biocare (Pacheco, CA, USA)
mAbDas-1	Barrett’s Esophagus	KM Das

*—ACS = adenoma carcinoma sequence; IBD’ = inflammatory bowel disease; ^—IM = Intestinal Metaplasia Marker. Cat# 555036 (BD Pharmingen(tm) Purified Mouse Anti-Human VEGF) is manufactured in Tatabanya, Hungary (BD Plant 2323).

**Table 2:** The significant clinical differences between the regressed and stable BE are contrasted.

Parameter (Number) Regressed Barrett’s Mucosa (10)	Stable Barrett’s Mucosa (36)	Probability
PlateletsX1000/cc ± sd 185.2 ± 27.4	249.4 ± 70.5	<0.003
Length BE initial (cm) 0.67 ± 0.43	1.45 ± 1.50	<0.016
Initial Inflammation x 0.071 ± 0.267	0.534 ± 0.534	<0.012

cc—cubic mm; x—mean; cm—centimeters; sd—standard deviation.

**Table 3:** Demographic data of patients.

Patient Parameters (*n*)	Regressed Barrett’s Mucosa (10)	Stable Barrett’s Mucosa (36)	Probability
Age (years ± sd)	52.9 ± 5.5	62.7 ± 0.22	<0.005
Sex (%Male:Female)	10:0 (100%)	33:3 (91.7%)	1
Ethnicity (%Black:White)	2:8 (20%)	10:24 (24.9%)	0.7
Body Mass Index	15.0 ± 2.9	29.1 ± 5.5	<0.01

sd: standard deviation.

**Table 4:** Data regarding BE and normal epithelium with and without inflammation.

Parameter	BE+ Infl BE− Infl	Normal Infl. Normal No Infl.		OR/*t*	*p* or CI
Serum Cr−	1.040 ± 0.185 1.409 ± 3.032	NSS NSS		*t*-Test	<0.022
ACE exposure	17/45 (78%) 160/95	(82%)		0.51	0.27–0.98
Urinary Cr−	115 ± 75 184 ± 100			*t*-Test	<0.022
Quan EtOH	13/20 (65%) 7/20 (35%)	NSS	NSS	6.86	<0.0002
Obese BMI > 28	7/23 (30%) 22/33 (67%)	NSS	NSS	0.22	<0.014
AA Ethnicity	8/38 (21%)	NSS	104/167(62%)	0.16	0.07–0.37
Dysplasia *	8/26 (31%) 9/39 (23%)	N/A	N/A		0.6

BE+—patients with BE; BE−—patients without BE; infl.—inflammation. OR—odds ratio; *p*—probability; CI—confidence interval; Cr−—creatinine; ACE—angiotensin converting enzyme inhibitors; *t*-test—Students *t* test; ETOH—ethanol intake; BMI—body mass index; AA—African American; NSS—not statistically significant; N/A—not applicable. *—none had high-grade dysplasia, but 5 patients had findings of indefinite dysplasia only in stable BE but with a significant difference at *p* < 0.046. BE was seen in 46 patients overall.

## Data Availability

The data presented in this study are available on request from the corresponding author due to Data Transfer Agreement. This term means a written agreement between the provider and the recipient of data that are transferred from one to the other. It defines what data may be used, how the data will be used, who may access and use the data, how the data must be stored and secured, and how the recipient will dispose of the data after completion of the research.

## Supplementary Materials

The following supporting information can be downloaded at: https://www.mdpi.com/article/10.3390/biom14091182/s1, Original images of Figure 11 can be found in supplementary materials.
