# Supplementary material for: Predicting Regression of Barrett’s Esophagus—Can All the King’s Men Put It Together Again?
Source: Biomolecules. 2024 Sep 20;14(9):1182. doi: 10.3390/biom14091182 (PMC11430295; doi:10.3390/biom14091182)

In figure 11 of Incremental time exposures to pH 4, expression at low pH does not appear to affect p87 expression.

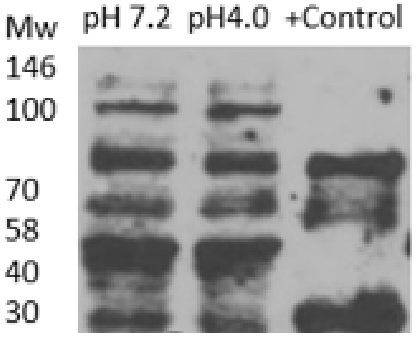

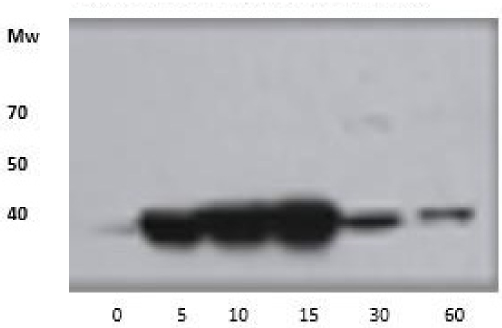

The physiological pH is 7.2 and the acidic pH is 4 and these exposures were all used for figures 11.

Figure 11C. At pH 4.0 0nly a 30min Exposure Evokes a Response with anti-p38$\alpha\beta$ on Left. Figure 11D On the Right is a Blot with anti-ERK as Shown on the Right-hand Side with an Maximal Response.

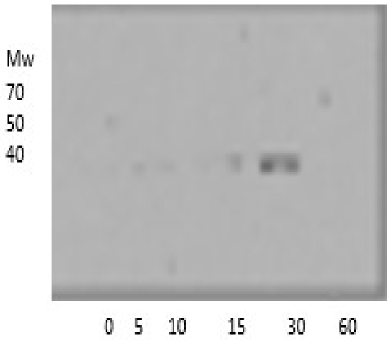

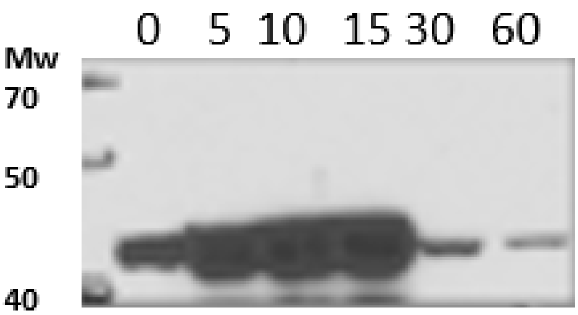

Figure 11E. Western blot of NCM460 Cell Lines Exposed to Different pH Levels for 1 hour, Stained for BGP Using the CEACAM1 Monoclonal.

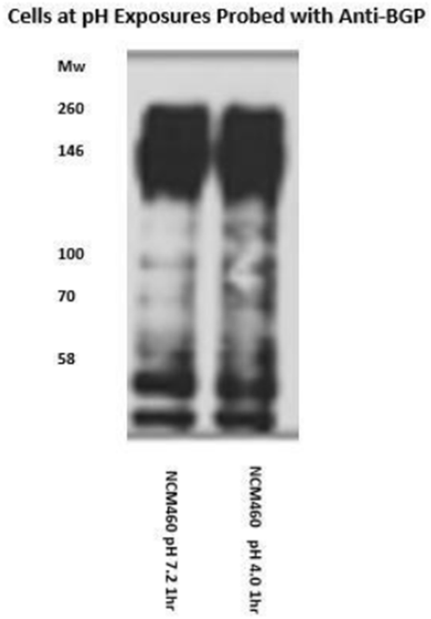

Supplement: Supplementary file 1 [file biomolecules-14-01182-s001.zip › biomolecules-3184776-SI.pdf]
